# Editorial: Executive Function and Education, volume II: Considerations for Academic Success - Across Contexts and Populations

**DOI:** 10.3389/fpsyg.2024.1365993

**Published:** 2024-01-29

**Authors:** Mariëtte Huizinga, Jacob A. Burack, Dieter Baeyens

**Affiliations:** ^1^Department of Educational and Family Studies, LEARN! Research Institute, Vrije Universiteit Amsterdam, Amsterdam, Netherlands; ^2^Department of Educational and Counselling Psychology, McGill University, Montreal, QC, Canada; ^3^Faculty of Psychology and Educational Sciences, KU Leuven, Leuven, Belgium

**Keywords:** executive function, academic success, individual differences, teacher-student relationship, culture, assessment, causality

Executive function is an umbrella term that refers to a set of fundamental cognitive functions essential for fostering goal-oriented and socially adaptive behavior. These cognitive functions enable us to flexibly retain crucial information, reflect within the context of novel situations, inhibit ingrained responses, and facilitate novel actions.

As the concept of executive function gained significant attention in the field of education, we initiated a Frontiers Research Topic on the intersection of executive function and education (Huizinga et al., 2018). In that Research Topic, the papers highlighted the critical role of executive function in academic success, illustrating how school performance is influenced not only by direct executive function engagement but also by the child's interactions with family members and teachers. These insights reflect the importance of incorporating context when developing educational interventions and curricula. Additionally, intervention research shows a shift toward more holistic strategies that are specific to context and/or individual differences.

In this second Research Topic, the role of executive function in academic success is extended to address ways that executive function relates to academic success across diverse groups by including scholarship on behavioral differences that impact achievement, including peer interactions, classroom emotion regulation, and learning disorders. With this focus in mind, we present four excellent contributions—two original research articles, one conceptual paper focusing on hypothesis and theory, and one systematic review.

The two empirical articles highlight the importance of examining the individual differences and the bidirectional and temporal relationships between executive function, teacher child relationships (TCRs), and academic performance. Southon evaluated whether characteristics of autism spectrum disorder (ASD), attention deficit hyperactivity disorder (ADHD), and developmental coordination disorder (DCD) can serve as indicators of academic success in college students, and examined if these traits influence the correlation between executive functioning and academic success. The participants were tasked with completing four self-administered questionnaires and provided their academic performance data based on their grades from university assignments. Whereas, ASD characteristics alone were not indicative of academic success, traits associated with ADHD and DCD were significant in moderating how executive function related to academic achievement.

Using a cross-lagged approach over three academic periods, Sankalaite et al. extended the well-established link between the quality of the TCRs, working memory, and academic performance to also include the bidirectional and time-related nature of these connections in primary school children and two-way interactions between working memory and academic performance. They found time-sensitive links between classroom working memory issues and subsequent TCR conflict, and a mutual influence between closeness in TCR and working memory challenges. A reciprocal relationship was also observed between arithmetic achievement and working memory difficulties. These findings highlight both benefits of interventions targeting improved TCR for children with working memory difficulties and that enhanced working memory can positively affect academic outcomes, thereby emphasizing the need for comprehensive educational strategies that address TCR together with working memory. Sankalaite et al. reveal that the bidirectional relationship between math and working memory only becomes evident when working memory is measured through questionnaires (i.e., perceptions by teachers and parents) and not through performance tasks (e.g., Corsi Block Tapping task). Questionnaires on executive function-related problem behavior probably also tap on other processes such as motivation or personality traits, where this is less the case for traditional performance task. A similar argument may hold for the predictive value of the Head-Toes-Knees-Shoulders (HTKS) task with respect to learning outcomes (see Kenny et al.). It is therefore safe to conclude on the one hand that the operationalization of executive function needs to be done thoughtfully, keeping the aim of the assessment well in mind. On the other hand, it shows the importance to consider executive function in the role of academic achievement while also not losing other educational processes out of sight.

Continuing with the topic of contextual factors that mediate the relationship between executive function and education, Cho et al. explored the widely recognized notion that East Asian immigrants' children often outperform their North American peers academically. Considering the crucial role of executive function in academic success and the faster development of executive function in East Asian cultures, Cho et al. posit that executive function disparities might explain the academic differences. They offer a new framework connecting executive function, culture, and academic performance, which incorporates contemporary theoretical perspectives on the essence of executive function and its connection to the social environment.

In attempting to establish the effectiveness of the HTKS task, a common early childhood self-regulation assessment, in predicting academic achievement through a comprehensive meta-analysis, Kenny et al. collated data from 69 peer-reviewed studies encompassing 413 effect sizes and nearly 20,000 children who met the research criteria. Their analysis revealed that the HTKS task reliably forecasted children's academic performance in areas such as literacy, spoken language, and math. It showed a particularly strong correlation with mathematical abilities over language and literacy skills, echoing previous studies' findings. The finding that the HTKS task is significantly linked to overall academic performance in children was consistent across varying participant characteristics and assessment methods, aligning with other meta-analyses on the connections between self-regulation, executive function, and academic achievement.

The papers in this second Research Topic invite us to reflect on the role of executive function on academic success. Although the importance of this relationship has been evidenced before, these new papers bring nuance to the debate and highlight challenges for research and daily educational practice. The findings from the papers indicate that the relationship between executive function and academic achievement is embedded in a larger system of processes. Holistic educational models like the Multilevel Supply-Use model by Brühwiler and Blatchford (2010) integrate theoretical and empirical insights to delineate factors that contribute to learning outcomes such as academic achievement. These factors are situated at the level of the supplier, notably the educational system, the school context, and classroom factors which are further differentiated into teacher characteristics (e.g., teacher competency), classroom context (e.g., SES), and classroom processes (e.g., TCR). Conversely, at the level of the user, the authors refer to learning environments (e.g., migration background), individual learning preconditions (e.g., executive function, ADHD, DCD) and individual learning processes (e.g., learning strategies). All factors at both levels are potentially interrelated in their prediction of learning outcomes. Already from a first glance at this model, we can conclude that the focus of this Research Topic is conceptualized as an association between individual learning preconditions (i.e., executive function) and learning outcomes (i.e. academic achievement) to which complexity is added in terms of moderating and mediating relationships of other factors.

Building on available evidence from this Research Topic and other literature, the causal nature of the relationship between executive function and academic achievement seems plausible. However, causality requires at least three conditions to be met: (1) change in executive function should be associated with change in academic achievement (i.e., association), (2) variation in executive function should precede its presumed effect in academic achievement (i.e., time order), and (3) variation of third variables should not lead to the association of executive function and academic achievement (i.e., non-spuriousness). We have ample evidence on the association between and time order of executive function and academic achievement, yet the Multilevel Supply-Use model challenges whether we can rule out all likely spurious effects on this relationship. The value of the papers in this second Research Topic is that they elucidate parts of such a spuriousness role of third variables (e.g., ADHD, DCD, ASD in Southon; TCR in Sankalaite et al.; cultural effects in Cho et al.), yet we are still left with considerable uncharted territory. We will illustrate this point with two examples. One, in a global world with more cultural diversity in classrooms, Cho et al. touch upon on the timely observation that we need to use a cultural lens to view the relationship among classroom process, executive function and academic achievement. Recent studies point to the cultural (dis)similarities of such concepts (e.g., Xu et al., 2023 on TCR), and suggest that insights from individualistic (predominantly Anglo-Saxon and Western-European) countries cannot be automatically applied to the dyadic relationship with a child from a collectivistic culture. Adopting a cross-cultural approach in studies on the topic will be needed to adequately deal with the inter-personal exchange between a teacher and students from diverse background and cultures in years to come. Similarly, the complex inter-relationship between teachers and students in the development of executive function (and consequently academic achievement) is further complicated by the influence of the child's other relationships, such as those with parents and peers, as well as by the complex interactions among these relationships (e.g., Vandenbroucke et al., 2017 for the interaction on parent-child and teacher-student relationship in the context of executive function performance). This type of ecological perspective will increase our understanding of the development of executive function, the impact on academic achievement, as well as the contextual influence on their relationship.

With a more comprehensive understanding of the relationship between executive function and academic achievement, the question arises how we can intervene to help improve academic achievement. Recent reviews (e.g., Mattera et al., 2021; Rowe et al., 2021) suggest that direct executive function training programs might not be effective in establishing durable and transferable skills. Therefore, more recent efforts are aimed at implementing classroom interventions in which executive function stimulation is complemented by training teachers in strategies to optimize dyadic TCR and classroom-level teacher-student interactions. These latest efforts prove to be (more) effective, although identifying which underlying mechanism exert their effect (i.e., mediators of the intervention) and whether improved post-intervention executive functioning and consequent higher academic achievement is the result of improved executive function skills or rather of reduced cognitive demands in an optimized learning environment (Sankalaite et al., 2021).

## Author contributions

MH: Writing – original draft, Writing – review & editing. JB: Writing – review & editing. DB: Writing – original draft, Writing – review & editing.
